# Endovascular treatment of a subclavian artery pseudoaneurysm in a patient with a pancoast tumor

**DOI:** 10.1186/s42155-025-00572-9

**Published:** 2025-06-18

**Authors:** Samuel J. Mouyal, Xavier Guerra, Tom Boeken, Alessandro Di Gaeta, Crina Bordeianu, Manuel Gargiulo, Olivier Pellerin, Marc Sapoval, Marc Al Ahmar

**Affiliations:** 1 Department of Vascular and Oncological Interventional Radiology, Assistance Publique-Hôpitaux de Paris, Hôpital Européen Georges Pompidou, Paris, France; 2 Université de Paris Cité, PARCC - INSERM Unité-970, Paris, France; 3 HeKA team, INRIA, Paris, France

**Keywords:** Pancoast tumor, Pseudoaneurysm, Hemoptysis, Subclavian artery, Endovascular interventional radiology, Stent graft

## Abstract

**Background:**

The authors report herein a rare case of an endovascular management of a giant subclavian artery pseudoaneurysm, revealed by a massive hemoptysis in a patient suffering from a Pancoast tumor.

**Case presentation:**

The endovascular procedure consisted of covering the subclavian artery rupture site with a stent graft after occluding the proximal segments of the ipsilateral internal thoracic and vertebral arteries.

**Conclusion:**

Subclavian artery rupture was effectively managed using endovascular techniques via radial access.

## Background

The Pancoast tumor, or superior pulmonary sulcus tumor, accounts for 3 to 5% of lung cancers [1]. Tumor invasion of the thoracic outlet structures over the lung apex can lead to Pancoast- Tobias syndrome, which is characterized by a constellation of ipsilateral symptoms, including neuralgic pain in the upper limb and Horner’s syndrome (miosis, ptosis, anhidrosis), due to the invasion of the brachial plexus (C8, T1, T2 nerve roots) and the sympathetic nerve chains (stellate ganglion), respectively [2–4]. When the subclavian vessels are invaded, it is not uncommon to observe stenoses or occlusions, which can result in manifestations of venous congestion or arterial ischemia of the limb. However, the occurrence of a subclavian artery (SCA) pseudoaneurysm caused by a Pancoast tumor appears to be atypical, as no cases have been described in the literature, which instead reports pseudoaneurysms of traumatic, iatrogenic [5, 6], infectious [7], and inflammatory [8] origins.

The authors report a case of endovascular management of a subclavian artery pseudoaneurysm, presenting with hemoptysis in a patient with a Pancoast tumor.

## Case presentation

The patient was a 36-year-old male with locally advanced large cell carcinoma of the right lung apex causing Pancoast-Tobias syndrome, under treatment for six months with radiation therapy and chemotherapy (Carboplatin, Pemetrexed, and Avastin).

**Fig. 1:**
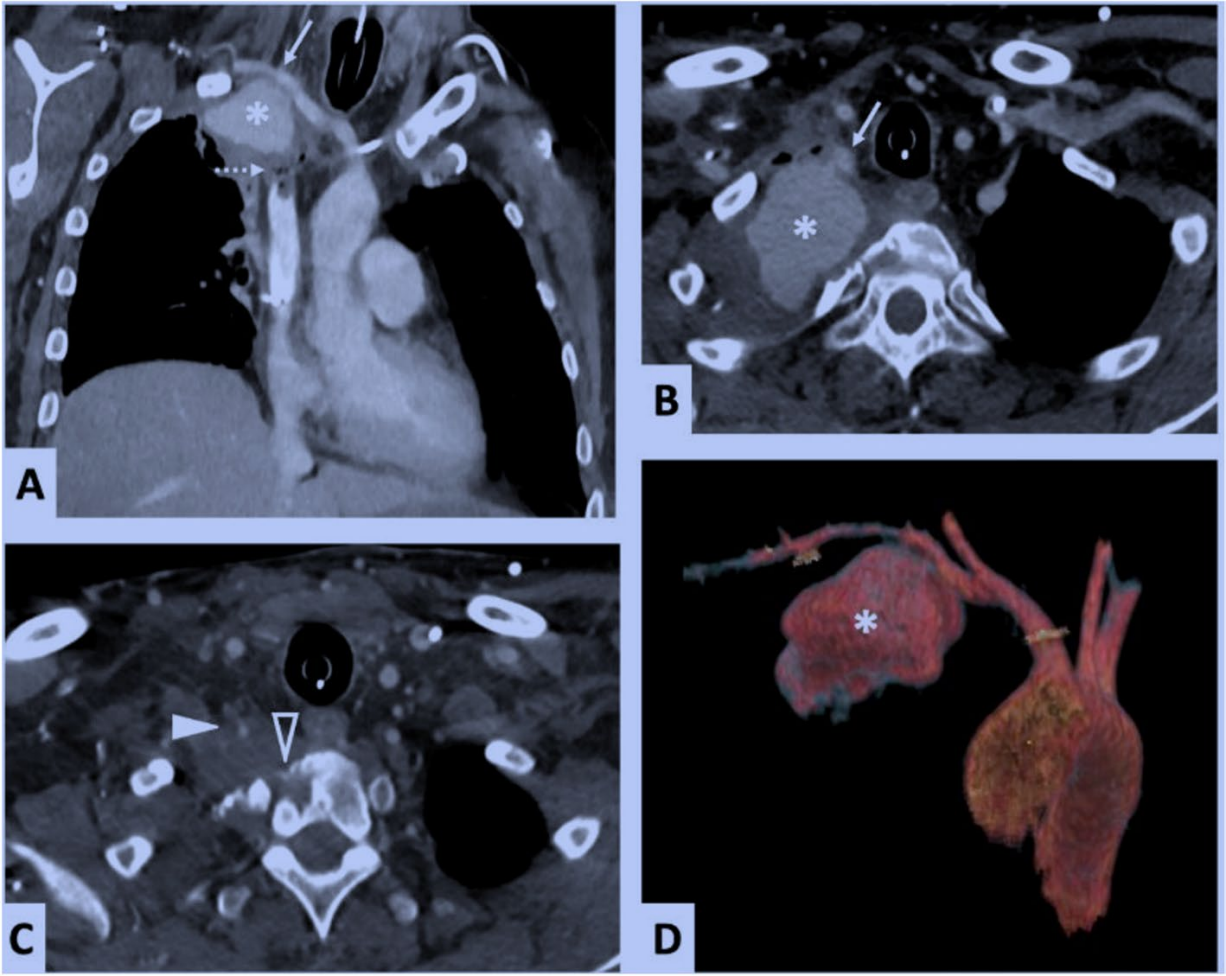
Thoracic CT angiography in the arterial phase after contrast agent injection, coronal slice (1-**A**), axial slices (1-**B** and 1-**C**), and volume rendering (1-**D**). Pseudoaneurysm of the right pulmonary apex (1-**A**, 1-**B** and 1-**D**, asterisk) contained by the edges of the Pancoast tumor, due to rupture of the posterior-inferior wall of the right SCA (1-**A** and 1-**B**, solid arrow). Tumor invasion of the mediastinum with thrombosis of the superior vena cava (1-**A**, dotted arrow), invasion of the right lateral part of the T1 vertebral body (1-**C**, hollow arrowhead), and encasement of the V1 segment of the right VA (1-**C**, solid arrowhead)

He experienced massive hemoptysis leading to hypoxia-induced cardiorespiratory arrest and was successfully resuscitated. After sedation, intubation, and hemodynamic stabilization, a contrast-enhanced chest CT scan (Fig. 1) revealed a 6 cm pseudoaneurysm originating from the right SCA. The pseudoaneurysm was contained by the margins of the excavated apical lung tumor. Moreover, the Pancoast tumor had eroded the right lateral part of the T1 vertebral body and encased the V1 segment of the ipsilateral vertebral artery (VA). After multidisciplinary discussion (intensive care physician, thoracic surgeon, and interventional radiologist), the patient was transferred by air ambulance to our center for emergency interventional radiologic treatment.

**Fig. 2:**
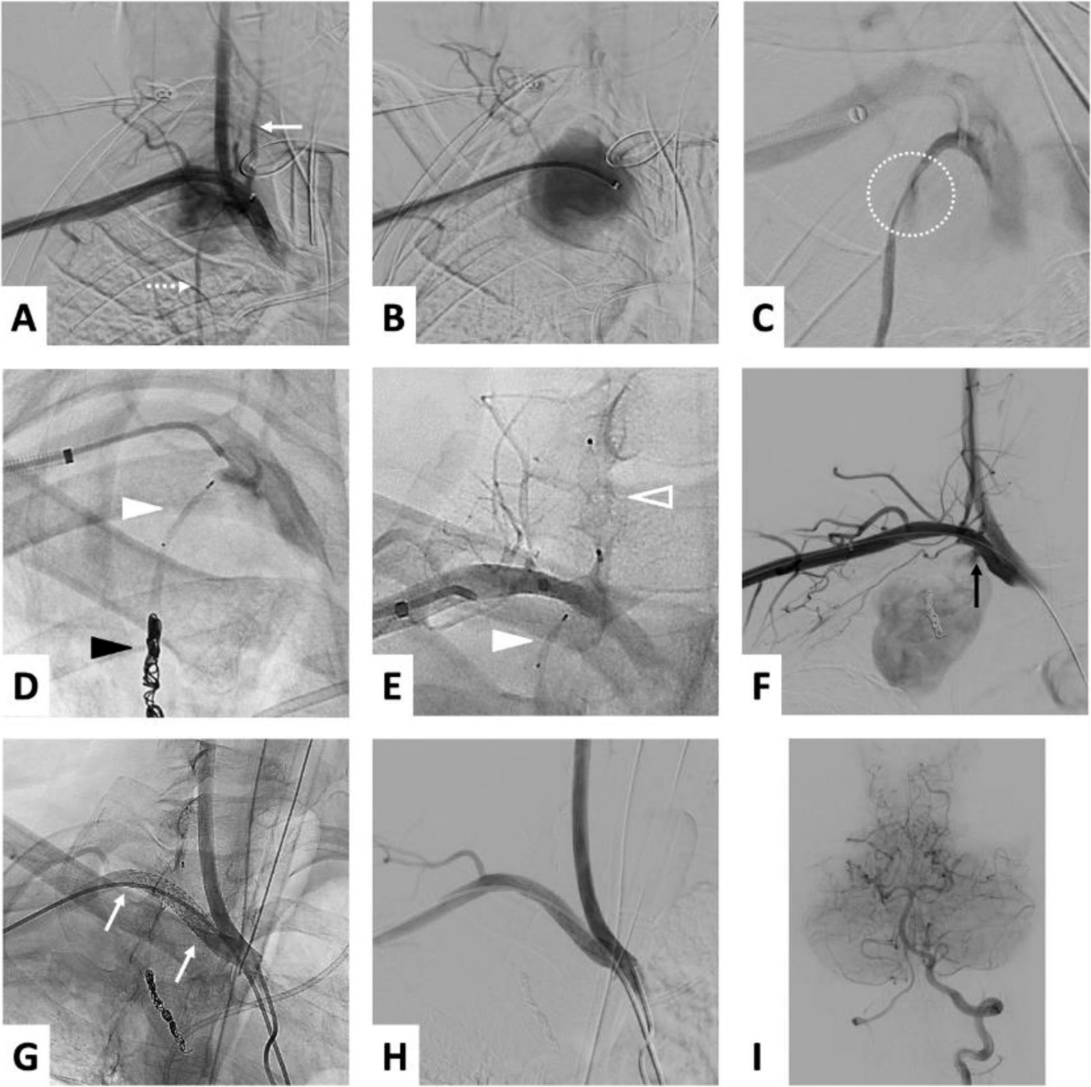
DSA of the right SCA showing pseudo-aneurysmal extravasation (with clarified angiographic projections for better identification of vertebral artery origin) (2-**A**: early phase, 2-**B**: late phase) in proximity to the right VA (2-**A**, solid arrow) and the right ITA (2-**A**, dotted arrow). Selective arteriography of the right ITA revealing extravasation from the proximal segment (2-**C**, dotted circle). Occlusion of the right ITA downstream of the rupture point with coils (2-**D**, black arrowhead) and with a micro-plug upstream (2-**D** and 2-**E**, solid white arrowhead). Occlusion of the V1 segment of the right VA with a plug (2-**E**, hollow arrowhead). Arteriography of the SCA after occlusion of the VA and ITA identifying the rupture point feeding the pseudoaneurysm (black arrow). Covered stent deployed in the right SCA (2-**G**, proximal and distal ends of the stent marked by white arrows) allowing complete exclusion of the pseudoaneurysm without residual extravasation (2-**H**). Angiography of the posterior cerebral circulation via the left VA showing no embolic complications (2-**I**). Images 2-**A** to 2-**D** were acquired in 20° right anterior oblique projection; images 2-**E** to 2-**H** were acquired in anteroposterior (frontal) view; image 2-**F **was acquired in frontal view with 15° cranial angulation

Intervention was performed using a right radial ultrasound guided approach via a 6-French (Fr) long sheath introducer. A digital subtraction angiography (DSA) of the right upper limb was conducted to safely navigate the angled diagnostic catheter to the SCA. DSA confirmed the perforation of the posteroinferior wall of the SCA a few millimeters upstream from the right vertebral artery ostium (Fig. 2A). Additionally, a perforation of the proximal segment of the internal thoracic artery (ITA) was identified. Both rupture points contributed to the pseudoaneurysm blood inflow (Fig. 2B).

Our strategy consisted of covering the SCA rupture site with a stent graft after occluding the proximal segments of the right ITA and VA. A prior assessment of the posterior cerebral circulation was performed by angiography, which demonstrated convergence of the vertebral arteries into the basilar trunk, with left vertebral dominance due to its larger diameter. The post-ostial segment of the right VA measured 4 mm and was occluded using a 6 mm Amplatzer™ Vascular Plug 4 (Abbott, North Chicago, Illinois, USA), delivered through a 0.038″ guidewire-compatible catheter. The ITA was then catheterized with a 2.7-Fr microcatheter (Progreat, Terumo, Tokyo, Japan) and embolized using the “sandwich technique”: coils (Azur CX-18, 6 × 20 mm, Terumo, Tokyo, Japan) placed downstream and an MVP™−5Q MicroVascular Plug (Medtronic) placed upstream of the rupture point, respectively (Fig. 2). The final step was the deployment of a 7 × 37 mm balloon-expandable stent graft (BeGraft peripheral, Bentley, Germany) centered on the SCA perforation and covering the ostia of the right VA, ITA, thyrocervical trunk and superior thoracic artery. The apposition of the stent graft to the vascular wall was optimized using an 8 mm diameter balloon. Complete exclusion of the pseudoaneurysm was confirmed on control DSA (Fig. 2). Additional angiography of the posterior cerebral circulation through the left VA demonstrated the absence of cerebral perfusion defects and retrograde perfusion of the right VA to the occluded proximal segment (Fig. 2). Hemostasis of the puncture site was obtained using a radial compression device (TR Band®, Terumo, Tokyo, Japan).

**Fig. 3:**
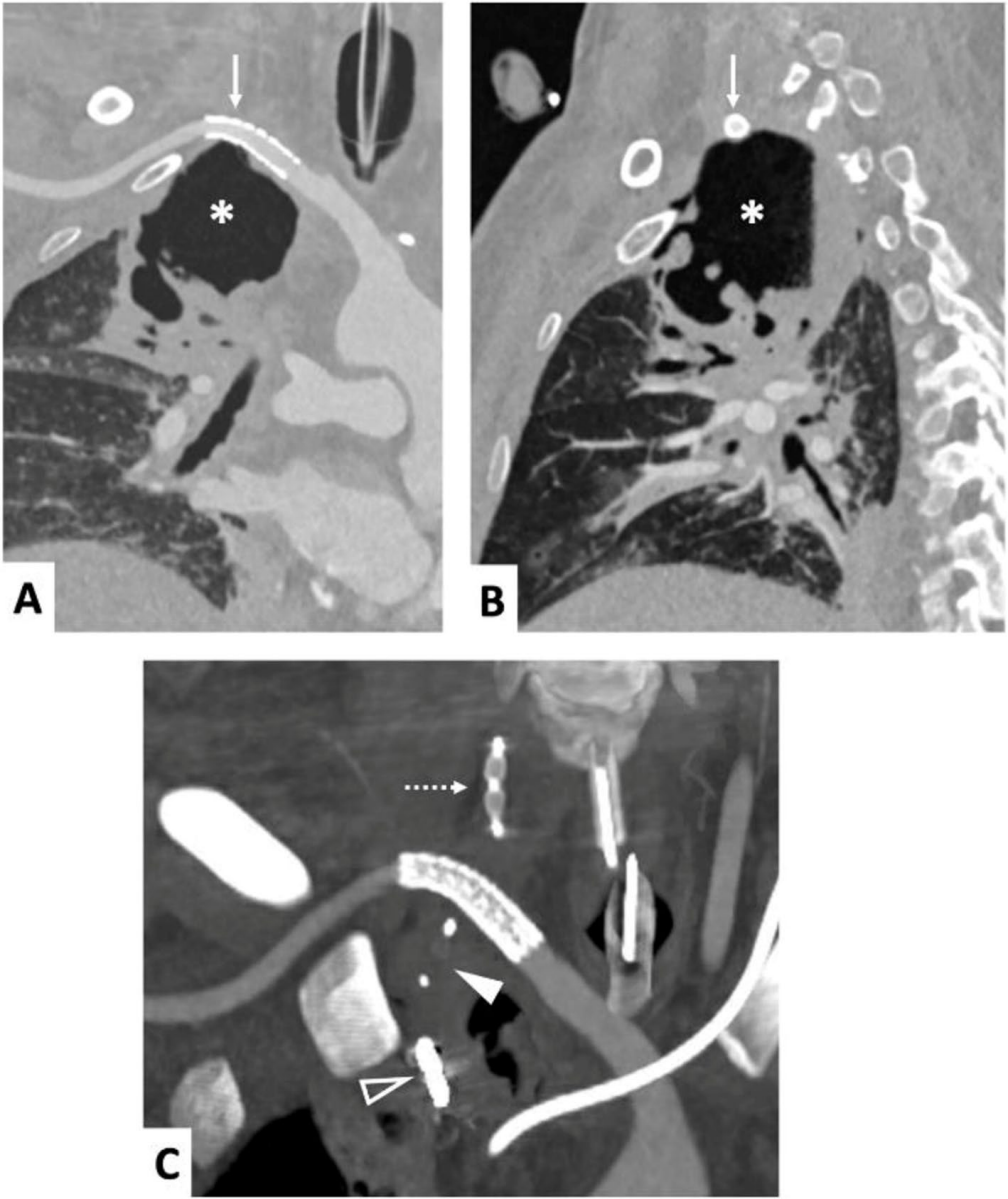
Chest CT-scan in the arterial phase after contrast agent injection, coronal (3-**A** and 3-**C**), sagittal view (3-**B**). Right SCA with regular caliber and patency of the stent graft (3-**A** and 3-**B**, arrow), adjacent to the upper pole of the right apical pulmonary excavation where the pseudoaneurysm was located (3-**A** and 3-**B**, asterisk). Occlusion of the proximal segments of the right ITA with a microvascular plug (3-**C**, solid arrowhead) and coils (3-**C**, hollow arrowhead), and of the right VA (3-**C**, dotted arrow)

A contrast-enhanced chest CT angiography at one week (Fig. 3) showed the patency of the right SCA, no endoleak and no active bleeding. The pseudoaneurysm has given way to a pulmonary excavation of the right upper lobe. The patient's fatal cardiac arrest three weeks later resulted from respiratory complications associated with prolonged mechanical ventilation and underlying pulmonary disease, without evidence of recurrent bleeding.

## Discussion

This case represents a rare subclavian artery rupture being effectively managed through endovascular intervention. This technique not only successfully controlled the acute hemorrhage but also maintained the integrity and patency of the vertebral artery, without any post-procedural complications. Considering the patient’s young age and ongoing curative chemotherapy and radiotherapy, with an anticipated life expectancy greater than one year without acute complications, an aggressive endovascular approach was deemed appropriate. This approach further demonstrates the practical application of minimally invasive techniques in addressing complex vascular emergencies associated with cancer, showcasing the evolving capabilities of endovascular interventions.

Endovascular treatment, in this instance, proved crucial for achieving hemostasis and ensuring the stability of nearby vascular structures through targeted embolization and the careful deployment of a covered stent. The success of this procedure highlights the significant role of refined interventional radiology techniques in addressing severe vascular challenges, particularly in patients with cancer, where traditional surgical interventions may pose heightened risks. The radial approach was chosen over the femoral route because it provided a more direct and proximal access to the subclavian artery, whereas the femoral approach would likely have been more technically challenging.

If vertebral angiography had revealed the left vertebral artery (VA) terminating as a posterior inferior cerebellar artery (PICA) without joining the basilar trunk (a scenario observed in ~ 6% of cases), occlusion of the right VA would not have been feasible due to the substantial risk of vertebrobasilar stroke [9]; treatment would likely have been abandoned.

Pancoast-Tobias syndrome is defined by the constellation of severe shoulder and arm pain corresponding with the distribution of the C8, T1, and T2 nerve trunks, alongside Horner syndrome—which includes ipsilateral ptosis, miosis, and anhidrosis due to disruption of the sympathetic nerve chain traveling to the head—and muscle atrophy in the hands.

In modern clinical practice, arterial complications directly caused by tumor invasion in Pancoast-Tobias syndrome are very rare. Advances in early detection and treatment, including chemotherapy and radiotherapy, have significantly reduced the incidence of such complications, which were more common in the past. Today, the more frequently observed vascular complication associated with thoracic tumors is superior vena cava syndrome, resulting from venous compression by the tumor.

Literature is poor in instances of tumoral complications of subclavian artery (SCA) treated by endovascular approach. Among these cases, there is the stenosis of the SCA by a lung cancer, causing ischemic disturbances in the distal fingers [2]. While surgical interventions for apical lung tumors may necessitate treatments involving the subclavian artery or lead to related complications [3, 4], there is no documented evidence of bleeding caused directly by tumor-induced rupture of the subclavian artery. This highlights a gap in the literature regarding such severe vascular events linked to tumor growth.

Current management of SCA injury is predominantly through endovascular approaches, particularly in post-traumatic, iatrogenic, and infectious scenarios, such as fungal infections and tuberculosis [5, 6, 10]. The first reported case of such endovascular treatment dates back to 1991 [7]. This technique has since been refined and widely adopted, offering a viable alternative to open surgery with benefits including minimized surgical risk and reduced recovery times [8]. There is one documented case of ruptured SCA aneurysm presenting as hemoptysis successfully treated using a covered stent [11]. However, this case was not of tumoral origin. A recent case review [10] expands knowledge on post-traumatic and iatrogenic pseudoaneurysms.

## Conclusion

In summary, this case marks a rare instance where a tumor-induced subclavian artery rupture was effectively managed endovascularly via radial access. It shows also the importance of vascular analysis for irregularities or pseudoaneurysm in apical tumors.

## Data Availability

All data generated or analyzed during this study are included in this published article.

## Abbreviations

DSA: Digital subtraction angiography
ITA: Internal thoracic artery
SCA: Subclavian artery
VA: Vertebral artery
